# Clinical placements as a challenging opportunity in midwifery education: A qualitative study

**DOI:** 10.1002/nop2.1139

**Published:** 2021-12-08

**Authors:** Maryam Modarres, Mehrnaz Geranmayeh, Mitra Amini, Monireh Toosi

**Affiliations:** ^1^ Department of Reproductive Health and Midwifery Faculty of Nursing and Midwifery Member of Nursing and Midwifery Care Research Center Tehran University of Medical Sciences Tehran Iran; ^2^ Department of Reproductive Health and Midwifery Faculty of Nursing and Midwifery Tehran University of Medical Science Tehran Iran; ^3^ Clinical Education Research Center Shiraz University of Medical Sciences Shiraz Iran; ^4^ Department of Reproductive Health and Midwifery School of Nursing and Midwifery Shiraz University of Medical Sciences Shiraz Iran

**Keywords:** clinical education, midwifery students, qualitative study

## Abstract

**Aim:**

The aim of this qualitative study was to explore some of the existing statuses of clinical education and examine the strengths and weaknesses of the clinical faculty programme.

**Design:**

A qualitative study.

**Methods:**

This qualitative study was designed through the directed content analysis approach, which was completed according to the Context, Input, Process, and Product evaluation model. The purposeful sampling method of maximum variation was employed to select 33 participants, and the data were collected through in‐depth individual interviews and focus group discussion.

**Results:**

After the interview transcripts were analysed, the participants' opinions of the clinical faculty programme and the relevant challenges, dimensions and factors were classified into four categories and sixteen subcategories. The categories were compiled in the CIPP evaluation model, including context, input, process and product. Based on the content analysis results, appropriate planning was recommended for teaching effective clinical skills to students.

## 1 INTRODUCTION

Midwifery education is a part of the higher education system that deals with human life, and attention to its quantitative and qualitative aspects is of special importance (Fasihi et al., 2004).

Midwifery training is done in different ways in different countries. In Iran, midwifery students are admitted to a four‐year university course via a national examination. The Ministry of Health and Medical Education has designed the midwifery training programme in a single curriculum for all universities across the country.

A statistically significant part of the midwifery course is devoted to acquiring clinical skills, during which students develop skills in groups of four to eight in clinical settings. To complete the midwifery course, students must participate in at least 60 natural deliveries, and in order to graduate, they must successfully pass a comprehensive midwifery examination.

The clinical training course is an opportunity to learn, acquire and develop clinical skills, during which students acquire the necessary practical skills for professional midwifery activities (Thompson et al., 2011). Clinical education provides an opportunity for students to translate theoretical knowledge into the necessary mental, psychological and motor skills for patient care (Omidvar et al., 2005). Graduates of this field must have acquired the minimum professional skills required for midwifery tasks (Wiegers et al., 2010).

Clinical education is considered one of the most important educational strategies in order to prepare students to enter clinical settings, take responsibilities, develop skills and improve the ability to decide on health issues (Birks et al., 2017).

In this regard, midwifery schools are responsible for graduating students who can provide midwifery services in accordance with the latest scientific advances (Cheraghi et al., 2019). On the other hand, pre‐planned improvement of clinical skills and knowledge of midwifery graduates can significantly enhance the quality of services provided at all levels of the health system. In addition to guiding students in practical and clinical fields, acquisition of clinical skills improves their knowledge and substantially enhances the quality of clinical education (Donough & Van Der Heever, 2018; Ekstedt et al., 2019; Graham et al., 2016).

However, the results of many studies in Iran have shown a relatively deep gap in midwifery students' theoretical and clinical education. Accordingly, existing clinical education does not convey to students the ability to achieve appropriate clinical competency (Sharghi et al., 2015). Evidence has also indicated that students' professional skills have decreased compared with the previous decade (Delaram et al., 2013; Mousavi & Montazeri, 2013). Accordingly, despite having theoretical knowledge, new graduates do not have sufficient skills and efficiency in clinical settings (Saleh et al., 2014). In other words, clinical education has not successfully achieved its goals of training skilled people and improving the quality‐of‐care services (Seyedamini et al., 2018).

In this context, various studies have demonstrated that multiple problems in clinical settings have prevented the achievement of educational goals (Valiee et al., 2013). Therefore, it seems that midwifery education needs fundamental changes in clinical education (Haghani & Hojat, 2013). In 2004, the standard programme for the midwifery profession stated that the training provided in the midwifery curriculum should ensure that midwifery students are prepared to practice the trade under the standards.

According to these standards, midwifery students must be able to provide clinical services on patients' bedsides. The educational goals were seen in the curriculum, and the theoretical and practical training must ensure these capabilities. In addition, midwifery students must have the necessary self‐efficacy to perform the assigned tasks (Bellack et al., 1998). Hence, the necessary skills have been developed in different countries to train midwifery students. The International Confederation of Midwives published the Minimum Standards for Clinical Midwifery Education in 2006 and 2008 (Butler et al., 2008). In the United Kingdom, the Nursing and Midwifery Organization has published professional standards for midwifery based on what midwives can do in their clinical careers (Leap et al., 2002).

Standard institutions in the United States have also extracted a list of essential clinical skills for midwives, including all the skills available in the midwifery curriculum, in particular pregnancy, obstetrics and gynaecology (Bellack et al., 1998).

Iran has also taken steps to coordinate and improve the quality of clinical education, including launching a clinical midwifery faculty programme. In this regard, since 2014, the plan to revive clinical education and establish faculty members in the midwifery department in hospitals and clinical settings was announced by the Ministry of Health and Medical Education to universities across the country.

The purpose of the midwifery clinical faculty programme is to return the midwife to her original position in performing natural delivery and to determine the position of midwifery professors and students in teaching hospitals. In this programme, midwifery faculty members who have the knowledge and skills of midwifery attend educational and medical centres and give scientific and high‐quality care for pregnant mothers (24‐hr stay in midwifery clinics in morning, evening and night shifts) and give clinical education for students in order to empower them in the fields of care, education and research.

The clinical midwifery faculty programme, as a new educational programme, needs continuous control and quality improvement. Evaluation and even revision of educational programmes fall into the field of educational evaluation. Since any educational programme is formed and developed through evaluation, a pivotal role must be given to the educational evaluation process.

Therefore, a constructive and effective evaluation model is expected to help decision‐makers and planners to make the right decisions throughout the development stages of an educational programme, including ideation, planning, design and execution (Fitzpatrick, 2011). The CIPP model is one of the models that can systematically evaluate an educational system. This model can be used to design and develop educational programmes and systems of management and accountability. Developed by Daniel Stufflebeam and colleagues in the 1960s, the CIPP evaluation model requires the evaluation of context, input, process and product in judging a programme's value (Stufflebeam & Zhang, 2017).

Emphasizing the constructive evaluation of the development stages of a programme (ideation, planning, design and execution), the CIPP evaluation model helps decision‐makers and planners to make the right decisions (Fitzpatrick, 2011). Considering the significance of promoting midwifery clinical education to achieve international standards, there is a need for studies to thoroughly evaluate the quality of midwifery clinical faculty programmes.

Since the clinical midwifery faculty programme has recently been performed in some Iranian universities of medical sciences, it seems that the authorities need to systematically eliminate the challenges of this clinical programme in qualitative studies in order to design optimal educational programmes to improve the quality of midwifery services provided for pregnant mothers. Hence, the researchers have decided to conduct this qualitative study on the clinical midwifery faculty programme based on the CIPP evaluation model to identify the challenges and propose solutions for promoting this programme and, ultimately, public health.

Since there has been no study on Iranian universities' clinical midwifery faculty programme, this study aimed to explore the themes related to the perception of the clinical midwifery faculty programme to provide helpful Information to policymakers that promote clinical education experiences.

## 2 METHODS

### 2.1 Aim and design

This is a qualitative study with a content analysis methods study, which aimed to explore some of the existing problems of clinical education and examine the strengths and weaknesses of the clinical faculty programme. The purposeful sampling method of maximum variation was employed to select 33 participants, and the data were collected through in‐depth individual interviews and focus group discussion.

To achieve the maximum variation, participants included all those who were somehow involved in the clinical faculty programme, age and different education were selected.

The study was conducted in the School of Nursing and Midwifery, Shiraz University of Medical Sciences, from August 2019–February 2020. This university (https://www.sums.ac.ir/en/home) enrols about 120 midwifery students and graduates about 30 midwifery students annually. Directed content analysis was used in this qualitative research to obtain rich and deep Information from the phenomenon under investigation (Speziale et al., 2011). Since qualitative research emphasizes trust, transparency, verifiability and flexibility, it is considered an excellent method to develop insight and interpretation in midwifery education (Polit & Beck, 2009).

### 2.2 Study participants

The present qualitative study with a content analysis approach was conducted using individual interviews and focus group discussion. Participants in the individual interviews included all those who were somehow involved in the clinical faculty programme at Shiraz University of Medical Sciences and included 11 midwifery instructors, eight midwifery staff, six obstetrics–gynaecology residents, five midwifery graduate students and three midwifery board officials who were selected based on purposeful sampling and were required to sign an informed consent form to enter the study. The inclusion criteria included participants who were somehow involved in the clinical faculty programme. The exclusion criteria included participants who did not participate in the midwifery clinical faculty programme or were not willing to participate in the study.

### 2.3 Data collection procedure

In order to determine the challenges of midwifery clinical education in dealing with the clinical setting, individual interviews and focus group discussions were used. Individual interviews with the participants were carried out in a face‐to‐face manner and in a convenient place at the School of Nursing and Midwifery, Shiraz University of Medical Sciences. Focus group discussions with midwifery students were also performed to achieve a deeper understanding of this phenomenon (Traynor, 2015). The participants were invited to take part in the research by telephone or email. The individual and group interviews were begun by asking the participants a general and open question about the description of their encounter with the clinical setting. Then, some other questions were asked based on the participants' statements and responses (Speziale et al., 2011).

The semi‐structured interviews and open‐ended guide questions were developed by the researchers based on the literature review and experts' opinions and included: “how do you feel about the clinical faculty programme in general,” “would you like to talk about those clinical experiences which you found most anxiety‐producing,” “what are the best and worst things that you think can happen during the clinical experience,” “how do you think clinical experiences can be improved,” “what is your expectation from clinical experiences” and “what are the benefits and disadvantages of clinical experiences in your opinion.” For clarification of the explanations, further probing questions were then asked (“could you give me an example” or “would you explain more”). The first two questions were general questions, which were used as icebreakers to stimulate discussion and put the participants at ease to encourage them to interact normally with the interviewer. The author conducted the interviews in Persian and then was translated them into English after all the analysis was finished.

After obtaining permission from the Ethics Committee of Tehran University of Medical Sciences, all interviews were conducted at the School of Nursing and Midwifery, Shiraz University of Medical Sciences. The participants were interviewed by the face‐to‐face method in the Persian language, and each interview was carried out in one session. In the focus group discussion, the students were encouraged to talk to one another, ask questions, exchange anecdotes and comment on each other's experiences and perspectives. Each interview lasted for 40–60 min. The focus group discussion also lasted for 85 min. Each of the interviews was digitally recorded and immediately transcribed after the end of the interview sessions. The interviews were continued with the participants until the data were saturated. In qualitative studies, data saturation indicates the completion of interviews, which means no new data are generated (Traynor, 2015).

### 2.4 Ethical considerations

The Ethics Committee approved the present study of Tehran University of Medical Sciences (IR.TUMS.FNM.REC.1398.057). After introducing herself to the participants, the researcher explained the study objectives to them and assured them about their information confidentiality and their authority to reject the invitation or remain in the study. She also requested the participants to sign written informed consent forms.

### 2.5 Data analysis

Data management was performed using MAXQDA 10 software. After each interview, the data were analysed through a directed content analysis approach proposed by Zhang and Wildemuth (Wildemuth, 2016). Directed content analysis was used in this research in order to identify and understand the midwifery students' challenges in dealing with the clinical setting. The qualitative data will be analysed using the content analysis method proposed by Zhang and Wildemuth (2016) in eight steps as follows. In the first step, the data will be prepared for qualitative content analysis. The recorded interviews will be transcribed, and the non‐verbal messages of the participants, such as tone of speech, silence and cry noted during the interview, will be added to the transcripts because the hidden concepts and patterns should be extracted from data.

The second step pertains to the analysis unit. The analysis or semantic unit is actually the most basic part of the text that is categorized and coded during content analysis. Therefore, the identification of these units is one of the most important and basic decisions of content analysis. In qualitative content analysis, personal themes including words, sentences and paragraphs are usually used. Therefore, one should seek the text for the phrases or expressions from which the themes are extracted.

The third step is to inductively categorize and code the themes through continuous comparison of the categories extracted from data. Similar codes will be grouped under a single subcategory, and then, the subcategories form the categories according to their relationship. The categories will be then organized in a way to achieve internal consistency and external inconsistency.

In the fourth step, the coding will be tested on a sample text. To this end, the researcher codes an excerpt, and then, two members of the research team will control the coding consistency. In the fifth step, after the agreement between the researcher and the two members of the research team on the coding consistency, a replicable process of coding will be generalized to the whole text.

The sixth step is to achieve coding consistency. To this end, the initial codes and their allocation to categories will be controlled once again by two other members of the research team and experts in qualitative research. This aims to eliminate any human error caused by fatigue or misunderstanding and changes in participants' perception of categories and coding rules over time that may lead to inconsistency because new themes and concepts may be extracted from the text.

The seventh step is to conclude the categorized and coded data. The features and dimensions of the categories will be detected, the connections between categories will be determined, the hidden patterns will be revealed, and the categories will be examined on a wide range of data so that the categories, subcategories and transcripts will be compared and the inputs and outputs will be extracted and investigated to show whether the main categories and themes represent the data. Finally, in the eighth stage, the formed categories will be reported (Wildemuth, 2016). Rethinking about the codes and the subclasses resulted in the extraction of four categories and sixteen subcategories. It should be emphasized that before the data were submitted to content analysis, the interview transcripts were sent to the participants to approve. All participants were satisfied with the interview transcripts and confirmed the results.

### 2.6 Trustworthiness of data

Credibility, dependability, conformability, transferability and authenticity were considered for the trustworthiness of the results (Polit & Beck, 2009). The credibility of the research results was strengthened by spending sufficient time on data collection, diversity of participants and member checking in which the transcripts and codes were returned to the participants for the clarification of any ambiguous codes. Dependability and conformability were enhanced through external checking and peer debriefing.

Therefore, the research team, including two expert qualitative researchers and two external supervisors, reviewed and rechecked the transcripts, codes and categories to find any conflicts in the coding process. Moreover, purposeful sampling was employed with maximum variation for transferability. Furthermore, authenticity was confirmed through member checking, and the participants compared the research team's results to their views. The interviews were conducted in Persian and were then translated into English for publication. Two different individuals translated the interviews into English, and a third person confirmed the translated versions.

## 3 RESULTS

The study participants included 11 midwifery instructors, eight hospital midwifery staff, six residents, five midwifery graduate students and three midwifery board officials. Almost 70 midwifery students also participated in seven focus group sessions. Due to the lack of time, one person refused to participate in the study (Table 1).

**TABLE 1:** Demographic characteristics of the study participants

Group participants	Number of participants	Age (years) Mean	Grade	Worked (years) Mean
Midwifery instructors	11	39.8	MSc or PhD	11.1
Midwifery staff	8	36.1	BSc or MSc	11.5
Obstetrics‐gynaecology residents	6	35.6	Physician	6.8
Midwifery graduate students	5	24	BSc	1
Midwifery board officials	3	53	PhD	26
Students participating in focus group	70	25.5	BSc	‐

After analysing the interviews with the participants about the challenges of midwifery students in dealing with clinical education, 1250 codes were extracted from the interview texts and were classified into the following four categories and sixteen subcategories. The categories were compiled in the form of the CIPP evaluation model and included context, input, process and product (Table 2).

**TABLE 2:** The categories and subcategories extracted from the data

Categories	Subcategories
Context	1. Necessity to implement the programme 2. Failure to achieve goals and implement regulations
Input	3. Inadequacy in the educational content 4. Heterogeneity of students in clinical skills 5. Lack of facilities and equipment 6. Professional competence of teachers 7. Inappropriate educational environment
Process	8. Active and interactive teaching 9. Training of professional midwifery requirements 10. Prominent presence of the instructor 11. Induction of professional identity to students 12. Restrictions on performing legal duties 13. Poor interactions among the medical staff 14. Failure in the management‐supervisory process
Product	15. Experienced graduates 16. A ground for promotion of community's health

### 3.1 Context

The context of the educational programme is to determine the effective elements in an educational environment and to identify the problems, needs and opportunities in an educational context. In the field of the educational programme, factors such as needs, facilities and problems in an educational environment are examined (Stufflebeam & Zhang, 2017). From the participant's perspective, the context included the necessity to implement the programme and failure to achieve goals and implement regulations.

#### 3.1.1 Necessity to implement the programme

According to the participants, the implementation of the clinical faculty programme was necessary in order to solve the existing problems in the clinical environment and integrate clinical skills. Regarding the necessity to implement the faculty programme, one of the participants said: “The presence of the professor can provide students with proper training. This means that the professor gives scientific training to students. During this program and with the presence of the professor, the students are more orderly and learn more scientifically. They learn more, and the implementation of this program has been essential for the scientific education of students” (45‐year‐old midwife, maternity hospital staff, individual interview).

#### 3.1.2 Failure to achieve goals and implement regulations

The success rate of any training programme is measured by the extent to which the programme achieves its goals. There are obstacles and problems in the faculty programme that have hindered the achievement of the educational goals of the programme. The study participants reported failures in achieving the goals and implementation of the regulations, such as poor information about the objectives of the training programme and incomplete implementation of the regulations. In this regard, one of the students stated: “Before entering the program, we were not taught anything about the goals. We read a series of theoretical articles and then went to the ward, but we do not know the goals of the program. We have not specified the educational goals of the program, and we do not know them exactly” (23‐year‐old midwifery student, 7th semester of midwifery, focus group discussion).

### 3.2 Input

The input of the clinical programmes includes all the factors that enter the training programme and pave the way for the implementation of the programme and achieving the goals of the programme. Input means all individuals and human resources, including students, professors, administrators, financial resources and scientific resources, that are included in an educational programme (Stufflebeam & Zhang, 2017). The five subcategories of the input of the clinical faculty programme from the perspective of the participants included inadequacy in educational content, heterogeneity of students in clinical skills, lack of facilities and equipment, professional competence of professors and inappropriate educational environment.

#### 3.2.1 Inadequacy in the educational content

In any educational programme, one of the most important educational indicators is the existence of desirable and effective educational content for teaching specific scientific or practical content. The results of the study showed that there was no desirable educational content in the clinical faculty curriculum. In this respect, one of the participants maintained: “We did not have written content, and the training of the instructors were not the same. For example, each instructor said something and taught the procedure in a different way; some instructors taught the procedures differently, and this would cause problems and confusion for us. The instructors themselves were challenged by this difference” (25‐year‐old student, 8th semester of midwifery, focus group discussion).

#### 3.2.2 Heterogeneity of students in clinical skills

Some participants believed that the students were not the same in performing clinical skills. In this regard, one of the participants stated: “Students' initial abilities vary when they enter the program; some students have better abilities, and some have lower abilities. We also have a student who is poor at performing clinical procedures. We should not evaluate all students together. In the end, all of them will reach an appropriate level of competence and acquire the necessary clinical skills during the training program” (Midwifery instructor, 36 years old, individual interview).

#### 3.2.3 Lack of facilities and equipment

One of the requirements of optimal education is the adequacy of educational and welfare facilities. An environment that is equipped with advanced educational facilities and desirable welfare ultimately leads to effective education and increases the students' interest. The results of the present study showed that the educational facilities of the programme were not at the desired level and that the lack of appropriate educational and welfare facilities had caused a decrease in the quality of the clinical education from the perspective of both students and educators. In this regard, one participant stated: “Well, we do not have a room for conferences. It means that the conference room space is not really suitable. Furthermore, we have a room where professors have to rest, for example, where there is a monitor and a small library. Educational equipment has affected and distorted education. The facilities are not enough to teach, and we do not even have a model for teaching students. We do not even have a video projector or something in the room that the students could work with” (Midwifery instructor, 39 years old, individual interview).

#### 3.2.4 Professional competence of instructors

Clinical instructors are one of the most important components of any clinical education and can have a profound impact on students' learning. Clinical instructors must have specific skills in communication and functional skills, clearly know how to apply these skills and be able to transfer them to students. In the current study, one of the strengths of the programme from the participants' viewpoints was the professional ability of the programme's instructors. In this regard, one of the participants said: “The professors had the required clinical skills and could transfer the skills to us. They also had the appropriate clinical ability and skills. They also had the necessary knowledge to provide scientific and practical materials related to midwifery. Their training was very useful to me” (26‐year‐old student, 8th semester of midwifery, focus group discussion).

#### 3.2.5 Inappropriate educational environment

One of the influential factors in any educational programme is the appropriate educational environment and background of that programme, which can have a statistically significant impact on the students' desires and interests. Regarding the inappropriate educational environment and context in the faculty programme, one of the participants mentioned: "From the beginning, we were supposed to have an independent ward with independent staff to teach students according to the midwifery rules, but until the ward is not separated and the medical environment is prevailing, the situation continues, and we do not have an independent environment for midwifery students. Working in the medical environment is very difficult, and midwifery students cannot be independent. It is not a suitable environment for education" (Midwifery instructor, 53 years old, individual interview).

### 3.3 Process

In the process dimension, implementation of educational programmes and determination of the impact of the educational programme on learners are discussed. In this area, the teaching‐learning activities and the management‐supervisory process are examined. Process refers to all the activities that take place during the implementation of educational programmes (Stufflebeam & Zhang, 2017). Based on the present study results, the process of the clinical midwifery faculty programme consisted of active and interactive teaching classes, teaching midwifery professional requirements, prominent presence of the teacher in education, showing professional identity to students, restrictions on performing legal duties, poor interactions of medical staff and failure in the management‐regulatory process.

#### 3.3.1 Active and interactive teaching

Active and interactive teaching is one of the essential skills in education, which is mainly related to teachers' educational skills. Students described active and interactive teaching as one of the pillars of an effective learning environment compared to teacher‐centred training. In this regard, one of the participants stated: “Clinical training methods actually depend on the instructor, but the program has the flexibility that each instructor can use different training methods according to the needs s/he sees. We have a computer system and an Internet search engine that can easily teach theory. We also have a good time with students to teach. In fact, there is the potential in the program that we meet the educational needs of the students according to their wishes and desires. According to the students' own opinions, we put the items that need more explanation in the educational planning and teach them in that field” (Midwifery instructor, 35 years old, individual interview).

#### 3.3.2 Training of professional midwifery requirements

Professional requirement training is an essential principle of midwifery care. Teaching ethics, midwifery rules and regulations, emergency care training and teamwork training are some of the highlights of the clinical faculty programme mentioned by the participants. In this regard, one participant said: “Yes, we were taught to respect the rights of the patients; i.e., maintaining patients' privacy, asking for permission from the patients, our behaviors towards non‐Iranian patients like Afghans, and following the ethical fundamentals regardless of the patients' nationalities or beliefs” (23‐year‐old student, 7th semester of midwifery, focus group discussion).

With regard to teaching midwifery rules and regulations in the programme, one of the participants mentioned: “I learned this training from the teacher, and I try to work legally. For example, I know that if I give medicine and I do not register it, there are consequences. They taught me the accurate process of registration. This program had a good thing, and the professors taught us the rules and regulations of midwifery in practice, we learned in practice that we had to follow the rules of midwifery” (24‐year‐old student, 8th semester of midwifery, focus group discussion).

#### 3.3.3 Prominent presence of the instructor

The instructor plays an educational role and a supportive role. This relationship leads to the formation of professional identity in students. The study participants noted the impact of the teacher's prominent role in clinical education. In this regard, one participant stated: “Instructors in this program focus in a very specific way on a student to teach her a specific skill, and this focus of the teacher on a student definitely affects her learning. Because the teaching involves an individual‐to‐individual process, I felt I had to learn this because I thought she was just teaching me, and I had to learn this. The subject was very important to me” (25‐year‐old student, 7th semester of midwifery, focus group discussion).

#### 3.3.4 Induction of professional identity to students

Professional identity includes the values and beliefs that guide individuals' thinking, actions and interactions with other people and plays a key role in the socialization of individuals. With the help of professional identity, a person establishes one's existence in one's profession and reaches excellence in the profession. Professional identity is formed by one's experiences in clinical settings. Regarding the induction of professional identity to students in the programme, one of the participants said: “Using new methods such as having a leader in the program has increased the students' motivation. They plan and run the program themselves; this participation is effective in increasing motivation in the program and gives students more motivation to work and study. In fact, the students find their job identity in this program” (Midwifery instructor, 39 years old, individual interview).

Regarding the increase of students' self‐confidence in the programme, one of the participants maintained: “The psychological support of the instructor in the program increases the students' self‐confidence, and we see that when students enter this program, their self‐confidence increases over time. They should and will gain higher self‐confidence during this program” (Midwifery instructor, 40 years old, individual interview).

#### 3.3.5 Restrictions on performing legal duties

Clinical environments are, in fact, the practical areas for students. In the present study, the participants noted barriers in the clinical settings that created constraints on the students' legal duties. In this regard, a participant maintained: “The personnel and residents intervene in performance of the procedures, which makes our work difficult and deprives us of our independence. Staff and residents come, and because of their haste in emptying the ward, they interfere with our legal duties and prevent us from doing our legal duties properly” (30‐year‐old midwifery instructor, individual interview).

#### 3.3.6 Poor interactions among the medical staff

Proper communication is one of the essential principles of midwifery and nursing care. The ability to interact appropriately is central to all nursing and midwifery activities. These interactions can be between the midwifery group and colleagues as well as between the midwifery group and patients. In this regard, the participants pointed out the weak interactions between the medical staff and the students. One of the participants said: “The work pressure of the staff was transferred to the students, and the staff did not do any work other than recording the birth events. The entire workload of nursing and midwifery is on the students, and the staff gave us all the unnecessary work not related to education. The staff did not cooperate with us well. They did not participate in teaching and did not answer our scientific questions. They also interfered with our teaching by giving unnecessary tasks to the students” (32‐year‐old student, 8th semester of midwifery, focus group discussion).

#### 3.3.7 Failure in the management–supervisory process

The management–supervisory process is one of the effective factors in promoting training programmes. In addition, continuous monitoring of training programmes identifies the weaknesses and enhances the strengths of each training programme. In the present study, the participants pointed out shortcomings in the management–supervisory process of the programme. In this respect, one of the participants said: “I did not see them coming from an office to oversee the program. When there was a problem, the faculty members came to discuss the problem in the program. However, I did not see them coming from any other ministry or organization for supervision. The supervision in this training program is not done seriously and academically” (34‐year‐old midwifery instructor, individual interview).

### 3.4 Product

The product of the clinical programme is related to evaluating and determining the effects of the educational programme on the graduates, the results of an educational programme compared to its goals, and the relationship between expectations and actual results. Output means all graduates, produced knowledge, and achievements of the training programme (Stufflebeam & Zhang, 2017). From the perspective of the current study participants, the product included experienced graduates and a ground for the promotion of the community's health.

#### 3.4.1 Experienced graduates

Clinical education is a dynamic process aimed at educating professionally qualified graduates. These graduates should be able to work independently and skilfully and apply the learned skills adequately in clinical settings. Regarding the professional competence of the graduates of the programme, one of the participants said: “I worked for a program, and I had the necessary ability when I entered the ward. I visited all the emergency cases, and the staff and the head of the ward were satisfied with my performance. The head of the department was satisfied with me at the beginning of the project and had a good view of me. I was even her substitute in the ward for a while when she was not the head nurse” (24‐year‐old graduate student, individual interview).

#### 3.4.2 A ground for promotion of community's health

Considering the impact of the programme on the community's health and creating a suitable environment for promoting the community's health, one of the participants maintained: “This program has an impact on increasing the level of women's health and, consequently, community's health. The continuous presence of students and instructors makes pregnant mothers calm. Students and instructors are involved in maternity care, and the services they provide are more accurate and can definitely improve the community's health” (26‐year‐old graduate student, individual interview).

## 4 DISCUSSION

Clinical education is a sensitive and important period in midwifery education for which there are different educational models used around the world. Identifying the problems associated with clinical, educational programmes and then resolving these problems will help us achieve educational goals, train skilful midwives, provide suitable care services and promote the quality of midwifery education. One of the requirements of optimal education is having an appropriate educational environment in terms of the equipment, professors (Mc Carthy et al., 2018).

Educational researchers believe that setting realistic goals that are fitted to facilities, especially at the beginning of the educational programme, is very effective in improving the quality of clinical education (Ghafourifard, 2016). Expressing goals facilitates the proper implementation of teaching–learning activities (Tavakoli et al., 2014).

In order to achieve the educational goals, it is necessary for teachers to clearly and accurately explain the goals of clinical internships to students and other educational groups at the beginning of their educational activities (Ghafourifard, 2016). The qualitative results of the present study showed that the participants believed that the programme was ineffective in achieving the goals and implementing the curriculum regulations.

Given the importance of achieving educational goals, it seems that the expression of goals and planning and implementation of programmes related to clinical education needs to be reconsidered (Fotoukian et al., 2013) because proper educational planning can increase the quality of clinical education and lead to the achievement of clinical education goals(Sharif & Masoumi, 2005).

In the current study, the majority of the students participating in the focus group discussions stated that the educational goals were not matched with the expectations of the ward staff. According to the researchers, formulating realistic goals and explaining these goals to the groups participating in educational programmes is very effective in improving the quality of clinical education and should be considered in developing clinical education programmes (Raisler et al., 2003).

One of the requirements of desirable education is the appropriateness of the educational environment in terms of physical space, facilities and equipment, professors and staff. In an environment where there is proper cooperation among different clinical groups, the realization of educational goals and ultimately the conditions for more effective education are provided (Elcigil & Sarı, 2007). The present study results demonstrated that the physical condition of the clinical education facilities was not optimal and that the lack of adequate facilities had reduced the quality of clinical education from the perspective of the students and their instructors.

Limited resources were also reported as a challenge in midwifery clinical training. Clinical training settings have always faced shortcomings. Admission of midwifery students based on the clinical capacity of the university, providing and improving welfare facilities appropriate to the educational needs of the departments and providing facilities for students to improve the quality of education is essential. Midwifery clinical training requires facilities and resources to be provided, which is consistent with the findings of similar studies conducted on the issue (Boelens et al., 2018).

In this context, identifying and resolving the shortcomings and deficiencies and preventing discrimination in the allocation of resources and facilities to medical and non‐medical students appear to be the main mission of academic training systems. Lack of independence of midwifery students in providing childbirth services and making decisions for low‐risk deliveries, lack of explanation about the position of midwifery students and description of midwifery duties in the clinical environment, disregarding students' opinions in clinical decisions, unnecessary interventions of female residents in natural childbirth and students' dissatisfaction with the method of clinical evaluation have also been mentioned as other weaknesses of clinical education.

Other Iranian studies have reported problems in clinical settings, as well. These studies indicated that the students were not satisfied with or had negative attitudes towards the facilities and equipment of clinical environments (Jooibari & Sanaghoo, 2010; Ramezani & Kermanshahi, 2011).

The participants in a qualitative study cited that such factors as insufficient facilities, educational centres burnout, the prevailing atmosphere on the patients' bedsides and students' lack of interest in their field hindered clinical learning. On the other hand, the availability of educational facilities caused students to acquire the necessary clinical competencies and to perform faster in emergencies. Since the clinical environment is a key place for nursing and midwifery students and is one of the factors affecting clinical education, it is very important to pay attention to it (Lambert & Glacken, 2004).

Moreover, providing clinical guidance, including internship objectives, departmental regulations, job descriptions of students and clinical instructors, time and method of evaluation and the resources needed by students at the beginning of clinical education, can promote the students' responsibility, knowledge of tasks and integration of theoretical learning with skills. From the perspective of the participants in the present study, the lack of clinical guidance in the educational programme has caused differences in the way of teaching clinical procedures, ultimately confusing the students in learning these procedures. Hence, the curriculum in this field of study should be developed in such a way that the objectives expected by the health system are met. In fact, ensuring the quality of the midwifery training programmes is crucial for the clinical training system.

The training needs of medical students and the professional goals of the training are considered components of the training based on the needs of the system (Olafsdottir et al., 2018). One of the effective factors in clinical education is the atmosphere of the educational environment, which includes the way of communication and the attitudes of the personnel and those involved in the educational environment (Atack et al., 2000).

One solution to this problem is to select experienced, expert and knowledgeable trainers (Rowe et al., 2012). Identifying the challenges of midwifery clinical training is the first step towards achieving midwives' educational and training goals.

The present study showed the clinical instructors efficient in improving midwifery clinical training as their most important responsibility. Therefore, the instructors should have or acquire the skills required for responding to the educational needs of the students (Raofi et al., 2009). From the participants' viewpoints, one of the strengths of this programme was that the trainers benefitted from appropriate clinical abilities.

In fact, by having characteristics such as effective communication, instructors transferred their knowledge and experiences to the students and were the bridge between theoretical and clinical education. In other words, the implementation of the teaching‐learning process through competent and efficient instructors could enable the students to make the most of their abilities. Clinical educators also have a tremendous effect on increasing the quality of clinical education and can make clinical experiences enjoyable for students (Peyman et al., 2011).

Instructors' motivation is mainly affected by professional autonomy in the areas of duties and professional support. The autonomy of midwifery instructors is necessary for developing trust, respect, skills and competency (Basaran Acil & Dinç, 2018). The factors reported affecting professional autonomy include transparency in the definition of the concept of professional autonomy, receiving support from managers, midwifery teamwork based on evidence and regular study, and using a comprehensive theory of professional autonomy (Balhara & Mathur, 2013; Laschinger et al., 2014).

In the present investigation, most of the participants were dissatisfied with the inappropriate treatment of the nursing staff, assignment of unrelated tasks to students and unnecessary interventions of female residents in natural childbirth. The way staff and other medical groups interact with students plays a key role in the students' clinical learning and can be a key factor in accelerating their learning process (Tang et al., 2005).

The majority of the participants in the current research reported the unfriendly behaviours of the physicians, staff and sometimes instructors towards the midwifery students. Unfriendly behaviours included verbal abuse, bullying and disrespect, and threatening of the students' health in clinical training settings (Dinmohammadi et al., 2014). Additionally, the clinical setting did not support the students and was referred to as the phenomenon of “vertical violence against students” (Hajihosseini et al., 2018).

The gap between theory and practice and the negative attitude of clinical staff towards clinical trainers may also be among the causes of inadequate clinical training.

Lukasse et al. showed that much clinical staff has a negative view of midwifery trainers who have not been in clinical practice for years or have not been updated. The old and outdated method of teaching leads to a gap between theory and practice (Lukasse et al., 2017).

A study in Ghana showed that environmental factors and interpersonal and academic relationships could negatively affect midwifery students, with interpersonal stressors being the strongest (Lukasse et al., 2017).

Midwifery students experience specific problems during their academic and clinical education, leading to uncertainty, dissatisfaction and failure to adapt to their profession (Carolan‐Olah et al., 2014).

Most of these problems stem from a wide range of potential issues in the clinical learning environment and interpersonal relationships that can affect student learning. While midwifery students usually have extensive knowledge, they do not have sufficient clinical skills and fail to apply their theoretical knowledge in a stressful environment (Cooper et al., 2012; Gönenç & Sezer, 2019).

Developing a safe environment and friendly behaviours in clinical settings can develop a sense of value in midwifery students and improve education and learning conditions. Induction of professional identity to the students was another concept identified in the present study. Having a professional identity is considered a pre‐requisite for achieving success in different fields of study. The steps required to be taken to reform the policies and clinical training programmes and eliminate or add new fields of study include the recognition of different factors associated with the students' attitudes towards their fields of study (Fida et al., 2018; Harris et al., 2013).

Mutual interaction with other medical groups shared understanding, and shared objectives are essential for creating positive student experiences in a learning environment (Thunes & Sekse, 2015). Another study seeking students' evaluation of certain factors in the clinical learning environment at a Slovakian university revealed that a positive attitude and an appropriate collaborative atmosphere improved the students' learning (Gurková et al., 2016).

Thus, the authorities in charge of the midwifery clinical training are expected to develop strategies to institutionalize beliefs and values according to the field of study and appropriate expectations to modify the physical conditions of the clinical environment in order to attract students and promote self‐esteem, motivation, professional identity and positive educational attitudes among midwifery students. Poor interaction among the medical staff was another concept identified in the present study. Educational support is one of the important components of clinical education in nursing and midwifery courses, which requires the interaction and cooperation of educational instructors and clinical nurses (Seidi et al., 2014).

The existence of appropriate professional interactions between students and medical staff helps the transition of students and leads to the formation of professional identity (Apker et al., 2006). Appropriate professional interactions also play an essential role in motivating students to learn, leading to a positive self‐concept and increasing their learning motivation (Andrews et al., 2006). Elo's study of students 'experiences in the clinical environment showed that poor communication between staff and students could lead to students' lack of interest in learning and their negative attitudes (Mikkonen et al., 2016).

In the present research, an inappropriate educational environment was reported as a challenge in midwifery training. It has been reported that the facilitating role of staff and physicians' cooperation with students contributes to the effectiveness of clinical education. Several Iranian researchers have referred to disrespect among the staff and the lack of coordination between the treatment and educational systems of the university (Reising et al., 2018).

The supportive behaviour of the staff can contribute to professional growth and affect the teaching–learning process (Jasemi et al., 2018). The supportive behaviour of the staff towards midwifery students and the intra‐organizational coordination among the faculties and hospitals appear to improve the status of the midwifery clinical training (Arimoto et al., 2012).

In the present study, the lack of a specific tool and method for evaluation was proposed as a problem in clinical education. The students had called for a revision of the method of evaluation. The existence of the same and specific evaluation method and its uniform application seems necessary for all students. In another study, Helminen found that the evaluation process of students' clinical practice lacked consistency. High diversity in assessment quality and differences in the mentors' perceptions of assessment forms were among the student assessment challenges (Helminen et al., 2016). According to researchers, one of the main challenges for educators in clinical education is evaluating students in clinical settings (McCutchan, 2015). Because evaluation can be a way to determine the achievement of goals and provide students with proper feedback, it seems necessary to modify and review the evaluation methods of the clinical faculty programme.

The present study revealed active and interactive teaching as a strength of midwifery training since efficient teaching methods are crucial for developing the skills required for students. As an objective of clinical training, developing basic skills involving problem‐solving and efficient communication can improve the quality of clinical training. In this context, the instructors' knowledge of modern teaching methods and the students' ability to make decisions was reported as the main clinical training strengths from the students' perspectives. Using active teaching methods in midwifery training can result in more profound learning and improve the students' clinical skills. Given the complexity of midwifery, using modern clinical training methods can enhance the level of clinical training. The present study identified a more comprehensive view of the problems in clinical education. In addition, the students emphasized their professional future, professional discrimination and the need for employing experienced instructors.

### 4.1 Limitations

As this study was qualitative, the obtained results cannot be generalized to other clinical education programmes. Lack of sufficient motivation among some of the participants to interview was one of the limitations of this study, and due to the limited resources, time and location and wide distribution of midwifery graduates, this study was unable to cover large groups of graduates.

## 5 CONCLUSION

Based on the results of the present study, the lack of appropriate physical and welfare space in the ward, lack of facilities for scientific conferences, intervention in clinical education, unnecessary intervention in the process of natural childbirth, lack of uniformity in clinical procedures and lack of a systematic evaluation system were among the essential problems of clinical education. Therefore, fundamental changes in clinical environments are necessary to improve clinical skills and achieve clinical education goals. The results of this study would help educators to design strategies for more effective clinical teaching. Hence, the findings should be considered by midwifery education professionals. Given the complexity of the field of midwifery, using modern clinical training methods, such as the clinical faculty programme, may enhance the efficiency of clinical training in Iran.

## CONFLICT OF INTEREST

The authors have no conflicts of interest relevant to this article.

## AUTHOR CONTRIBUTIONS

Maryam Modarres, Mehrnaz Geranmayeh, Mitra Amini and Monireh Toosi performed conceptualization of the study, coordination, acquisition of data and drafting of the manuscript. All authors read and approved the final manuscript.

## ETHICS APPROVAL AND CONSENT TO PARTICIPATE

Written informed consent forms were obtained from all participants. This study was approved by the Ethics Committee of Tehran University of Medical Sciences, Tehran, Iran (code: IR.TUMS.FNM.REC.1398.057).

## Data Availability

The data are available on request from the corresponding author.
